# Blockade of PAR2 Signaling by Punicalagin as a Therapeutic Strategy for Atopic Dermatitis

**DOI:** 10.3390/ijms26188920

**Published:** 2025-09-13

**Authors:** Hyejin Jeon, Yohan Seo, Wook-Joo Lee, Yunkyung Heo, Won-Sik Shim, Wan Namkung

**Affiliations:** 1 College of Pharmacy and Yonsei Institute of Pharmaceutical Sciences, Yonsei University, 85 Songdogwahak-ro, Yeonsu-gu, Incheon 21983, Republic of Korea; isy0803@naver.com (H.J.); ddukdae12@gmail.com (Y.S.); ykheo107@naver.com (Y.H.); 2 Department of Bio-Nanomaterials, Bio Campus of Korea Polytechnics, 112 Dongan-ro, Ganggeong-up, Nonsan 32943, Republic of Korea; 3 College of Pharmacy, Gachon University, 191 Hambangmoero, Yeonsu-gu, Incheon 21936, Republic of Korea; onlynice20@gachon.ac.kr

**Keywords:** punicalagin, PAR2, antagonist, atopic dermatitis, itch

## Abstract

Atopic dermatitis is a chronic inflammatory skin disorder characterized by persistent inflammation and severe pruritus. Current anti-inflammatory agents carry risks of long-term adverse effects, while antihistamines provide limited relief of pruritus. Protease-activated receptor 2 (PAR2) has emerged as a critical mediator of both inflammation and pruritus, representing a promising therapeutic target. In this study, we investigated the therapeutic potential of punicalagin (PCG), a potent PAR2 antagonist, in atopic dermatitis. PCG fully and potently inhibited trypsin-induced PAR2 activation in HaCaT cells with an IC_50_ of 1.30 µM, exhibiting over 40-fold greater selectivity over PAR1. PCG significantly inhibited PAR2-induced phosphorylation of ERK1/2 and NF-κB in both HaCaT and human dermal fibroblast cells and reduced IL-8 secretion in HaCaT cells. In addition, PCG did not significantly affect other pruritus-related GPCRs including H1R, H4R, TGR5, 5HT2A, 5HT2B, and MRGPRX2 at 30 µM. Notably, PCG strongly blocked PAR2-AP-induced scratching in mice. In addition, PCG improved skin lesions, reduced dermatitis severity scores, and alleviated scratching behavior in a DNFB-induced atopic dermatitis model. These effects were associated with reduced epidermal thickness, decreased serum TSLP levels, and inhibition of PAR2-dependent calcium signaling in dorsal root ganglion neurons. These findings demonstrate that PCG is a selective PAR2 antagonist that effectively alleviates both inflammatory and pruritic symptoms of atopic dermatitis, suggesting its potential as a novel therapeutic agent.

## 1. Introduction

Atopic dermatitis is one of the most common chronic inflammatory skin diseases, affecting up to 20% of the global population. It is characterized by severe pruritus, dry skin, erythema, and lichenification, all of which significantly impair patients’ quality of life [1,2]. Moreover, atopic dermatitis can trigger the development of other allergic conditions such as asthma and allergic rhinitis, underscoring the severity of the disease and the critical importance of effective treatment [1].

The pathophysiology of atopic dermatitis involves complex interactions among genetic predisposition, immune dysregulation, and skin barrier dysfunction [3,4]. Impaired barrier function allows allergen and microbial invasion, leading to excessive activation of keratinocytes and immune cells that release diverse inflammatory mediators, including Th2 cytokines and thymic stromal lymphopoietin (TSLP). These mediators amplify immune responses and promote disease persistence [5]. These mechanisms contribute to impaired epidermal barrier function, increased sensitivity to environmental antigens, and severe pruritus that provoke scratching behavior. This establishes the characteristic “itch-scratch cycle” of atopic dermatitis, in which repetitive scratching exacerbates skin barrier disruption and continues the inflammatory cascade [6].

Therefore, current therapeutic approaches for atopic dermatitis primarily target inflammation through topical corticosteroids and calcineurin inhibitors as first-line treatments [7]. However, long-term use of these therapies may cause adverse effects, including skin atrophy and increased susceptibility to infections. Although recently developed biologic therapies have shown promising clinical outcomes, they are expensive and require injectable administration [8,9]. Furthermore, because atopic dermatitis-associated pruritus involves both histamine-dependent and histamine-independent mechanisms, antihistamines offer only limited symptomatic relief. These limitations emphasize the urgent need for safer, more effective, and more accessible therapeutic options [10].

To address these limitations, attention has recently turned to alternative molecular targets involved in both inflammation and itch. Among them, protease-activated receptor 2 (PAR2) has emerged as a promising candidate [11,12]. PAR2 is a G protein-coupled receptor (GPCR) and a member of the PAR family, which includes PAR1, PAR2, PAR3, and PAR4. These receptors are uniquely activated via N-terminal cleavage by extracellular proteases [13]. In particular, PAR2 is selectively activated by trypsin. When activated, PAR2 participates in diverse physiological and pathological processes, including inflammation, immune response, and sensory transmission [14]. In skin inflammation, PAR2 is involved in keratinocyte activation, cytokine secretion by immune cells, and sensory nerve signaling [15].

Accumulating evidence from preclinical and clinical studies strongly implicates PAR2 in the pathophysiology of atopic dermatitis [16,17]. Mice overexpressing PAR2 spontaneously develop characteristic atopic dermatitis features, including dry skin, intense pruritus, and eczema-like lesions, suggesting that PAR2 plays a critical role to induce the disease hallmarks [15,18]. Furthermore, clinical studies have demonstrated increased serine protease activity and elevated PAR2 expression in lesional skin relative to non-lesional skin in patients with atopic dermatitis, providing translational evidence supporting a pathogenic role for PAR2 [16,19]. Notably, PAR2 mediates pruritus through histamine-independent pathways, representing a key contributor to chronic and treatment-refractory itching in atopic dermatitis. Therefore, PAR2 represents a promising therapeutic target that can address both inflammation and itching [16,20,21].

Here, we assessed the therapeutic potential of punicalagin (PCG), a recently identified novel PAR2 antagonist [22], in atopic dermatitis. We evaluated the antagonistic activity of PCG in human skin-derived cell lines, followed by in vivo assessment of its effects in a PAR2-activating peptide (PAR2-AP)-induced pruritus model and a 2,4-dinitrofluorobenzene (DNFB)-induced atopic dermatitis model. PCG demonstrated promising anti-inflammatory and anti-pruritic activities.

## 2. Results

### 2.1. Selective and Potent Inhibition of PAR2 by PCG in HaCaT Cells

**Figure 1:**
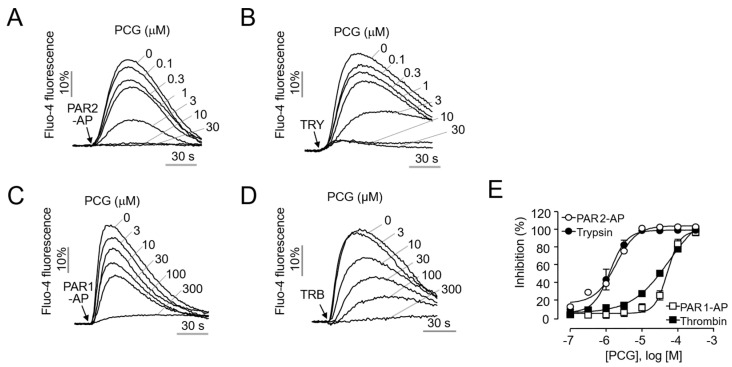
Potency and selectivity of PCG for PAR2. (**A**,**B**) Inhibition of PAR2-mediated calcium mobilization by PCG in HaCaT cells. Cells were pretreated with PCG for 10 min before stimulation with 30 µM PAR2-AP or 30 U/mL trypsin (TRY). (**C**,**D**) Effect of PCG on PAR1-mediated calcium mobilization in HaCaT cells. Cells were pretreated with PCG for 10 min before stimulation with 30 µM PAR1-AP or 30 U/mL thrombin (TRB). (**E**) Summary of dose–response curves for PAR2 and PAR1 inhibition by PCG.

To evaluate the PAR2 antagonistic effect of PCG, experiments were conducted using HaCaT cells, a human keratinocyte cell line that endogenously expresses PAR2. PCG demonstrated potent inhibition of PAR2-AP induced calcium mobilization with an IC_50_ value of 1.89 µM (Figure 1A) and effectively suppressed trypsin-mediated PAR2 activation with an IC_50_ of 1.30 µM (Figure 1B). Given the high sequence homology between PAR1 and PAR2 [23], the effect of PCG on PAR1 activation was also investigated. PCG inhibited PAR1-AP-induced activation with an IC_50_ of 51.88 µM and thrombin-induced activation with an IC_50_ of 58.75 µM (Figure 1C,D). These results indicate that the IC_50_ values for PAR2 inhibition are >27 fold lower than those for PAR1, demonstrating the markedly higher selectivity of PCG for PAR2.

### 2.2. PCG-Mediated Inhibition of PAR2-Induced ERK1/2 and NF-κB Phosphorylation and IL-8 Release in HaCaT Cells

**Figure 2:**
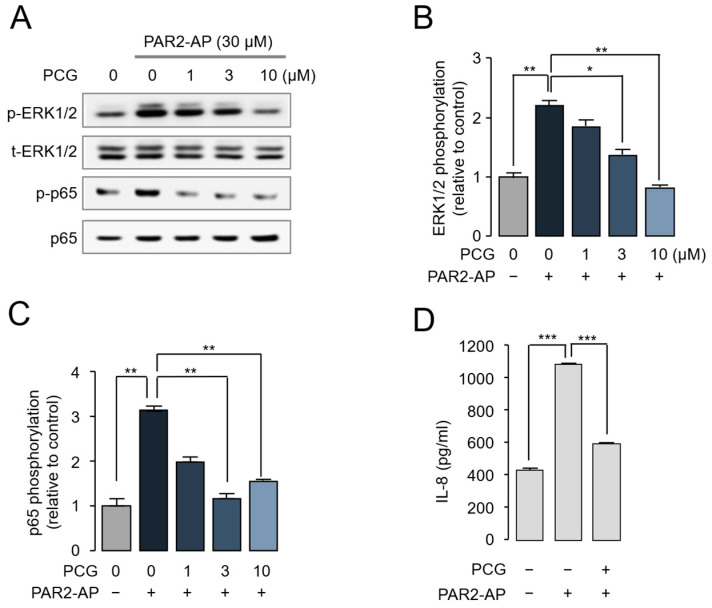
PCG suppresses PAR2-induced inflammatory signaling in HaCaT cells. (**A**) Representative immunoblot showing the effect of PCG on ERK1/2 and p65 phosphorylation. HaCaT cells were pretreated with various concentrations of PCG for 20 min before stimulation with 30 µM PAR2-AP. (**B**,**C**) Quantification of phosphorylated ERK1/2 and p65 levels. Phosphorylated ERK1/2 bands were normalized to total ERK1/2 and phosphorylated p65 (p-p65) was normalized to total p65. (**D**) Effect of PCG on PAR2-AP-induced IL-8 secretion. HaCaT cells were pretreated with PCG for 0 min and then stimulated with 30 µM PAR2-AP for 6 h. IL-8 levels in cell culture supernatants were measured by ELISA. Data are expressed as mean ± SEM (*n* = 3). * *p* < 0.05, ** *p* < 0.01, *** *p* < 0.001.

Phosphorylation of ERK1/2 and the NF-κB subunit p65 plays an important role in atopic dermatitis by regulating skin inflammation, barrier dysfunction, and the production of proinflammatory mediators [24,25]. To examine the effect of PCG on PAR2-induced ERK1/2 and NF-κB phosphorylation, HaCaT cells were stimulated with PAR2-AP. As shown in Figure 2A–C, PCG significantly inhibited PAR2-mediated phosphorylation of ERK1/2 and p65 in a concentration-dependent manner. IL-8 is a potent pro-inflammatory cytokine that recruits and activates neutrophils and other immune cells, and its expression is markedly increased in atopic dermatitis lesions [26]. To assess the effect of PCG on PAR2-mediated IL-8 production, IL-8 levels in the culture medium were quantified by ELISA. PAR2-AP stimulation markedly increased IL-8 secretion, which was substantially reduced by PCG treatment (Figure 2D).

### 2.3. PCG-Mediated Inhibition of PAR2-Induced ERK1/2 and NF-κB Phosphorylation in Human Dermal Fibroblasts

**Figure 3:**
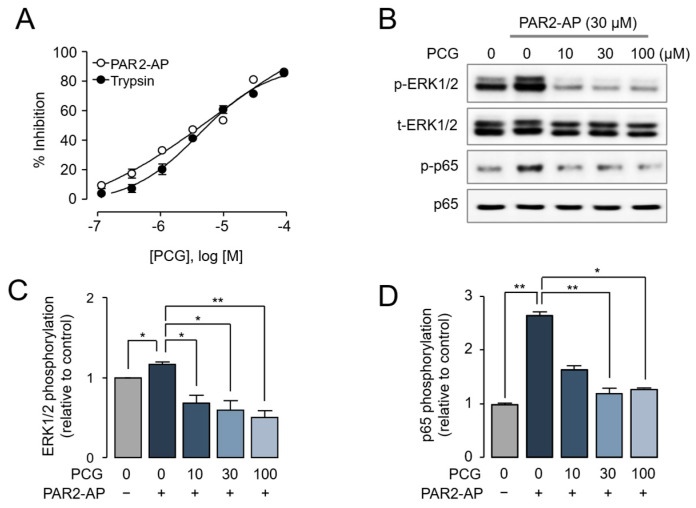
PCG inhibits PAR2-mediated signaling pathways in human dermal fibroblasts. (**A**) Dose–response curve of PCG on PAR2-mediated intracellular calcium levels in HDFs. Cells were pretreated with various concentrations of PCG for 10 min before stimulation with 30 µM PAR2-AP or 30 U/mL trypsin. (**B**) Representative immunoblot demonstrating the inhibitory effect of PCG on PAR2-AP-induced phosphorylation of ERK1/2 and p65 in HDFs. Cells were pretreated with various concentrations of PCG for 10 min, followed by co-treatment with 30 µM PAR2-AP for 5 min. (**C**) Quantification of phosphorylated ERK1/2 levels. Phosphorylated ERK1/2 bands were normalized to intact ERK1/2. (**D**) Quantification of phosphorylated p65 (p-p65) levels, normalized to total p65. Data are expressed as mean ± SEM (*n* = 3). * *p* < 0.05, ** *p* < 0.01.

Human dermal fibroblasts (HDFs) are key structural cells of the dermis that contribute to skin homeostasis, wound healing, and inflammatory responses, and are known to express functional PAR2 [27]. We further evaluated the effect of PCG on PAR2 activation and PAR2-mediataed ERK1/2 and NF-κB signaling in HDF cells. As shown in Figure 3A, PCG strongly inhibited PAR2-AP-induced intracellular calcium increase with an IC_50_ value of 7.09 µM. PCG also suppressed trypsin-mediated PAR2 activation with an IC_50_ of 4.06 µM. The effect of PCG on PAR2-induced phosphorylation of ERK1/2 and NF-κB was examined by immunoblotting. PCG significantly suppressed PAR2-mediated phosphorylation of ERK1/2 and p65 in HDF cells in a concentration-dependent manner (Figure 3B–D).

### 2.4. PCG-Mediated Suppression of PAR2-AP-Induced Scratching in BALB/c Mice

**Figure 4:**
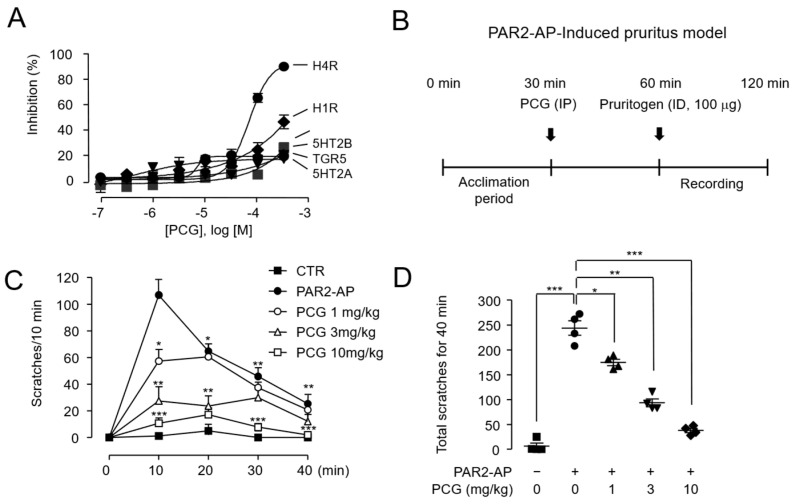
Selectivity of PCG for itch-related GPCRs and its effect on PAR2-AP induced scratching in BALB/c mice. (**A**) Effect of PCG on the activity of the pruritus-related GPCRs. Cells overexpressing each GPCR were pretreated with PCG for 10 min before stimulation with their respective agonists. (**B**) Experimental timeline for PAR2-AP-induced pruritus model. (**C**) Time course of scratching behavior. Scratching behavior was recorded at 10 min intervals for 40 min post-injection. (**D**) Cumulative scratching bouts over 40 min post-injection. The graph shows the dose-dependent effect of PCG compared to vehicle control. Data is presented as mean ± SEM (*n* = 4 mice per group). * *p* < 0.05, ** *p* < 0.01, *** *p* < 0.001 compared to PAR2-AP with vehicle group.

PAR2 is a well-established mediator of histamine-independent pruritus pathways and represents a critical therapeutic target for overcoming antihistamine-resistant itching [20]. Prior to in vivo studies, we evaluated the selectivity of PCG against pruritus-related GPCRs including H1R, H4R, TGR5, 5HT2A, 5HT2B, and MRGPRX2 using appropriate functional assays. As shown in Figure 4A, PCG exhibited no significant inhibitory activity against any of these receptors at 30 µM. Subsequently, we evaluated the antipruritic efficacy of PCG using a PAR2-AP–induced scratching mouse model (Figure 4B). PCG dose-dependently reduced PAR2-AP-induced scratching behavior (Figure 4C,D). Notably, at the highest tested dose (10 mg/kg), scratching behavior was markedly reduced to levels comparable with untreated controls. These results indicate that PCG effectively suppresses PAR2-mediated pruritus.

### 2.5. PCG Attenuates DNFB-Induced Atopic Dermatitis in Mice

**Figure 5:**
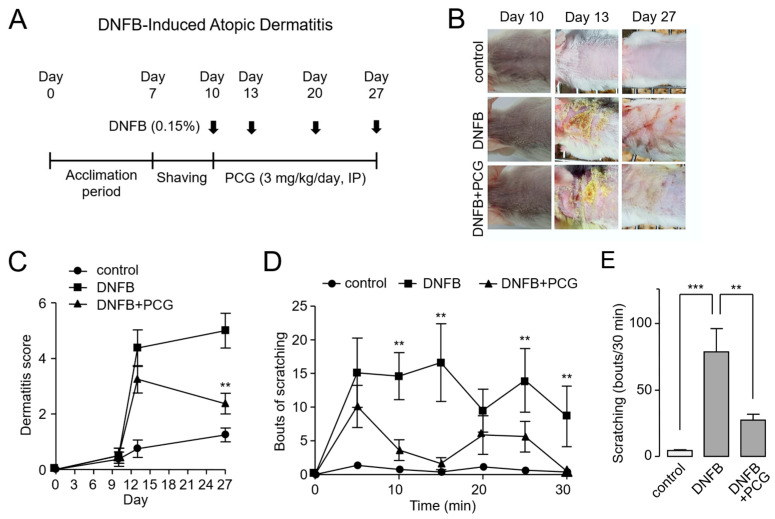
PCG alleviates DNFB-induced atopic dermatitis like lesions in mice. (**A**) Experimental timeline for DNFB-induced atopic dermatitis model. (**B**) Representative images of back skin show improvement in erythema and edema in the PCG-treated group compared to the DNFB-only group. (**C**) Dermatitis severity scores over the experimental period. (**D**) Time course of scratching behavior measured at 10 min intervals for 30 min following the final DNFB challenge. (**E**) Cumulative number of scratching bouts recorded over the 30 min observation period. Data are presented as mean ± SEM. ** *p* < 0.01, *** *p* < 0.001 compared to DNFB group (*n* = 5–6 mice per group).

We investigated the therapeutic potential of PCG using a DNFB-induced mouse model of atopic dermatitis. DNFB is known to reproduce atopic dermatitis-like symptoms such as skin inflammation, pruritus, and increased epidermal thickness through repeated exposure [28]. Mice were acclimated for one week prior to sensitization. On day 7, the dorsal skin was shaved, and 0.15% DNFB was applied four times on days 10, 13, 20, and 27. To evaluate its therapeutic efficacy, PCG (3 mg/kg) was administered daily via intraperitoneal injection from day 10 to day 27 (Figure 5A).

The therapeutic effects of PCG were evaluated in several aspects. In DNFB-treated mouse skin lesions, severe atopic dermatitis-like lesions were observed. In contrast, mice treated with PCG showed markedly improved skin appearance, with enhanced skin condition (Figure 5B). These observations were supported by dermatitis severity scores, which showed a considerable reduction in the PCG-treated group compared to the DNFB-only group (Figure 5C). Given that pruritus represents a cardinal feature of atopic dermatitis, we next examined scratching behavior over a 30 min observation period in 5 min intervals. The DNFB group exhibited the highest frequency of scratching, whereas PCG treatment significantly reduced this behavior (Figure 5D). Similarly, the total cumulative number of scratches during the observation period was markedly lower in the PCG-treated group, further supporting its antipruritic effect (Figure 5E).

### 2.6. PCG Mediates Therapeutic Mechanisms in DNFB-Induced Atopic Dermatitis

**Figure 6:**
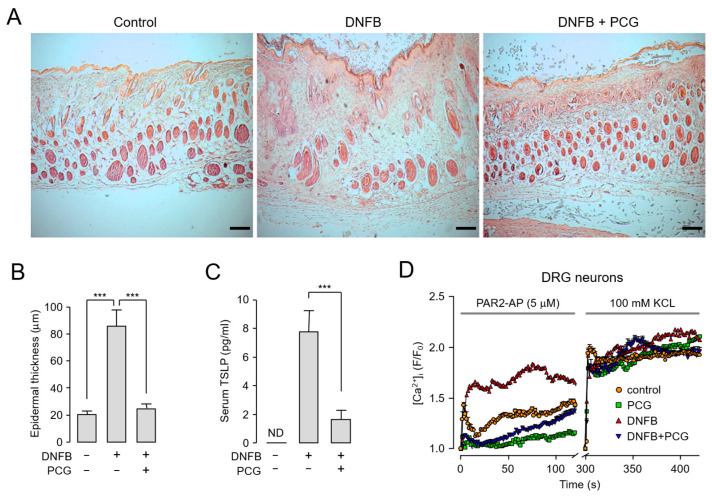
PCG normalizes epidermal hyperplasia, inflammatory markers, and neuronal hypersensitivity in DNFB-induced atopic dermatitis. (**A**) Representative H&E-stained skin sections showing epidermal thickness on day 28. Scale bar = 100 µm. (**B**) Quantitative analysis of epidermal thickness measured from H&E-stained sections (*n* = 5–6 mice per group). (**C**) Serum TSLP levels measured by ELISA. ND, not detected (*n* = 5–6 mice per group). (**D**) Calcium imaging of dorsal root ganglion (DRG) neurons in response to PAR2-AP (5 µM) stimulation. Traces show [Ca^2+^]_i_ changes over time, with KCl (100 mM) used as a positive control for neuronal viability (*n* ≥ 20 per group). Data are presented as mean ± SEM. *** *p* < 0.001 compared to DNFB group.

To further elucidate the therapeutic mechanisms of PCG in atopic dermatitis, we assessed epidermal thickness, serum TSLP levels, and calcium reactivity in dorsal root ganglion (DRG) neurons. Atopic dermatitis is characterized by increased epidermal thickness resulting from excessive keratinocyte proliferation, which contributes to inflammation and skin barrier dysfunction [29]. As expected, hematoxylin and eosin-stained (H&E-stained) skin tissue revealed a marked increase in epidermal thickness in DNFB-treated mice compared to controls. Notably, this thickening was significantly attenuated in the PCG-treated group, with measurements comparable to those of the control group (Figure 6A,B). Consistent with these histological improvements, we also observed changes in inflammatory cytokine expression. TSLP, a keratinocyte-derived cytokine central to the immune response in atopic dermatitis [30], was substantially elevated in the serum of DNFB-treated mice. However, this increase was effectively suppressed by PCG treatment (Figure 6C). We next investigated whether PCG modulates sensory neuron excitability by analyzing calcium responses to PAR2 activation in DRG neurons. DNFB exposure led to a considerable increase in PAR2-AP-induced calcium influx relative to controls. While PCG alone had no effect on calcium signaling, co-treatment with DNFB and PCG significantly inhibited the DNFB-induced calcium response. In contrast, PCG did not inhibit calcium responses in DRG neurons stimulated with 100 mM KCl, indicating selectivity of this effect (Figure 6D). Taken together, these findings indicate that PCG alleviates atopic dermatitis through a multifaceted mechanism involving the restoration of epidermal morphology, suppression of proinflammatory cytokines, and normalization of neuronal sensitivity.

## 3. Discussion

Emerging evidence highlights PAR2 as a promising therapeutic target in atopic dermatitis, due to its pivotal role in skin barrier impairment, inflammatory cytokine release, and type 2 immune responses [15,17]. While PAR2 antagonists such as PZ-235 have demonstrated in vivo efficacy in mouse models of atopic dermatitis [31], their clinical translation has been limited by poor stability, suboptimal oral bioavailability, and formulation hurdles. These limitations underscore the urgent need for novel PAR2-targeting approaches that offer superior pharmacological and therapeutic profiles [32]. In this study, our comprehensive investigation identifies PCG as a potent PAR2 antagonist with substantial therapeutic potential, highlighting its promise as a candidate for effective atopic dermatitis therapy.

PCG modulates both HaCaT and HDF cells, leading to comprehensive inhibition of PAR2-driven pathways, including intracellular calcium mobilization, ERK1/2 and NF-κB phosphorylation, and IL-8 release (Figure 1, Figure 2 and Figure 3). These findings highlight its capacity to regulate PAR2 signaling across multiple skin cell types pivotal to atopic dermatitis pathogenesis [15,31,33]. Moreover, because PCG inhibits both calcium signaling and ERK/NF-κB pathways downstream of PAR2 activation, it is expected to exhibit greater therapeutic efficacy for atopic dermatitis compared to PAR2 antagonists such as GB88, which exhibit pathway selective inhibition [34].

Pruritus in atopic dermatitis remains a therapeutically intractable symptom, as antihistamines have demonstrated limited efficacy across multiple clinical trials [10], underscoring the need to target non-histaminergic pruritus pathways. Among these pathways, PAR2 functions as a key mediator by activating sensory neurons through TRPV1 and TRPA1 channels [35,36,37]. In the present study, PCG dose-dependently suppressed PAR2-AP-induced scratching in mice (Figure 4C,D), and the DNFB-induced increase in calcium signaling by PAR2 activation was markedly suppressed in DRG neurons from PCG-treated mice (Figure 6D). Furthermore, PCG showed minimal antagonistic activity against other pruritus-related GPCRs (H1R, H4R, TGR5, 5HT2A, 5HT2B, and MRGPRX2), even at a high concentration of 30 µM (Figure 4A). These results suggest that PCG can selectively inhibit PAR2, indicating its ability to specifically suppress PAR2-mediated pruritus in atopic dermatitis. Thus, PCG has the potential to effectively attenuate both inflammation and histamine-independent pruritus in atopic dermatitis.

The suppression of epidermal hyperplasia and reduction in serum TSLP levels by PCG treatment in the DNFB-induced atopic dermatitis mouse model (Figure 6) suggest that PCG can attenuate TSLP level by suppressing PAR2-mediated inflammatory signaling. Particularly, given that TSLP is a pivotal upstream cytokine that initiates Th2-driven inflammation and epithelial barrier disruption [5,30], PCG is expected to effectively suppress early immunological events in atopic dermatitis. Taken together, the inhibition of PAR2 activity by PCG presents a novel therapeutic strategy for atopic dermatitis. In this proof-of-concept study, intraperitoneal administration was chosen as an appropriate route to demonstrate efficacy; however, we recognize that oral or topical administration would ultimately be more clinically relevant given the chronic and localized nature of atopic dermatitis. Therefore, future preclinical studies incorporating these administration routes will be essential to more accurately evaluate the therapeutic potential of PCG.

PCG is a natural compound with antioxidant and anti-inflammatory properties [38], and at higher concentrations it may potentially elicit off-target effects. For this reason, in the DNFB-induced atopic dermatitis mouse model we selected a dose of 3 mg/kg to better reflect PAR2 selectivity. Although off-target effects of PCG cannot be completely excluded in this study, our findings nonetheless demonstrate that PCG exerts therapeutic effects on atopic dermatitis, at least in part, through inhibition of PAR2 activity.

As a naturally derived polyphenolic compound from *Punica granatum*, PCG presents several pharmacological advantages compared to synthetic PAR2 antagonists. For instance, AZ3451, the most potent synthetic antagonist reported to date, failed to demonstrate anti-inflammatory efficacy following oral administration. This lack of efficacy is largely attributable to poor bioavailability, a limitation common to many small-molecule PAR2 antagonists [39]. In contrast, PCG exhibits several properties that directly address these limitations. Its long history of dietary consumption in humans confers a well-established safety profile, while its documented oral bioavailability and favorable tolerability support its feasibility for chronic administration in long-term inflammatory disorders [40,41]. Taken together, these attributes highlight PCG as a promising candidate to overcome the pharmacokinetic and safety barriers that have hindered the clinical translation of previous PAR2-targeted agents, offering a promising therapeutic approach for conditions such as atopic dermatitis. Nevertheless, potential off-target actions cannot be completely excluded, and comprehensive preclinical investigations, including long-term safety and pharmacokinetic evaluations, are essential to fully support PCG’s therapeutic development.

In conclusion, PCG exhibited potent PAR2 antagonistic activity and effectively attenuated both inflammatory responses and PAR2-dependent pruritus in a mouse model of atopic dermatitis. By inhibiting PAR2-mediated intracellular calcium mobilization and ERK/NF-κB signaling, PCG alleviates skin inflammation and pruritus, highlighting its potential as a therapeutic candidate for atopic dermatitis.

## 4. Materials and Methods

### 4.1. Cell Culture and Cell Lines

HaCaT cells were cultured in high glucose DMEM (Welgene Inc., Gyeongsan, Republic of Korea), and HDF cells were cultured in low-glucose DMEM (Welgene Inc., Gyeongsan, Republic of Korea). All media were supplemented with 10% FBS, 100 U/mL penicillin, and 100 µg/mL streptomycin, and all cells were grown at 37 °C, 5% CO_2_. All cells were obtained from the Korea Cell Line Bank (Seoul, Republic of Korea).

### 4.2. Materials and Reagents

PAR1 activating peptide (PAR1-AP, TRLLR-NH_2_) and PAR2 activating peptide (PAR2-AP, SLIGRL-NH_2_) were synthesized by Cosmogenetech Co., Ltd. (Seoul, Republic of Korea). AZ-3451 and Substance P were purchased from Tocris Bioscience (Bristol, UK). trypsin, thrombin, PCG, and lithocholic acid were obtained from Sigma-Aldrich (St. Louis, MO, USA). Serotonin and histamine were purchased from Tokyo Chemical Industry (Tokyo, Japan).

### 4.3. Intracellular Calcium Measurement

HaCaT and HDF cells were seeded in 96-well clear bottom plates and cultured overnight. Cells were loaded with Fluo-4 dye (Invitrogen, Carlsbad, CA, USA) in assay buffer for 1 h according to the manufacturer’s instructions. Following dye loading, cells were pretreated with various concentrations of PCG (dissolved in 1% DMSO) for 10 min. Intracellular calcium mobilization was then induced by the addition of PAR2-AP, trypsin, PAR1-AP, or thrombin. Fluorescence was measured using a FLUOstar Omega microplate reader equipped with two reagent injectors (BMG Labtech, Ortenberg, Germany).

### 4.4. Immunoblotting

For Western blotting, HaCaT and HDF cells were cultured overnight in 6-well plates with serum-free medium. The following day, cells were treated with the appropriate compounds and protein samples were prepared as previously described. Total protein (40 µg) was loaded into each well and separated using 4–12% Tris-glycine precast gel (KOMA BIOTECH, Seoul, Republic of Korea). The separated proteins were transferred to PVDF membranes and blocked with 5% BSA in phosphate-buffered saline containing 0.1% Tween-20 (PBST) for 1 h. Membranes were incubated overnight at 4 °C with primary antibodies: anti-phospho-p65 (Cell Signaling, Danvers, MA, USA), anti-p65 (Santa Cruz Biotechnologies, Santa Cruz, CA, USA), anti-p42/44 (Cell Signaling, Danvers, MA, USA), and anti-phospho-p42/44 (Cell Signaling, Danvers, MA, USA). After washing three times with PBST, membranes were incubated with appropriate HRP-conjugated secondary antibodies for 1 h at room temperature. Following three additional washes, proteins were detected using the ECL Plus immunoblotting detection system(Fusion Solo, Vilber Lourmat, Marne-la-Vallée, France).

### 4.5. Cytokine Release (IL-8, ELISA)

IL-8 levels were assessed using a human IL-8 ELISA kit (Abcam, Cambridge, UK) according to the manufacturer’s guidelines. The assay procedure was performed as follows: 50 µL of sample and blank control were dispensed into 96-well microplates and incubated for 1 h at room temperature. Wells were subsequently washed three times using the provided wash buffer. Next, 50 µL of 1× biotinylated antibody solution was added to each well and incubated for 30 min. Following this incubation step, TMB substrate solution was applied and allowed to develop for 10 min. The reaction was stopped by adding 100 µL of stop solution to each well. Absorbance readings were obtained at 450 nm using an Infinite M200 microplate reader (Tecan, Männedorf, Switzerland).

### 4.6. PAR2-AP-Induced Pruritus Model in BALB/c Mice

Six-week-old male BALB/c mice were acquired from DBL (Chungcheong-do, Republic of Korea) and acclimatized to laboratory conditions for one week. On the experimental day, mice were allowed a 30 min adaptation period before receiving an intraperitoneal injection of PCG. After 30 min, PAR2-AP (SLIGRL-NH_2_) was administered via intradermal injection into the dorsal neck region. Scratching behavior was then observed and recorded for 40 min. A bout of scratching was defined as a series of continuous hind limb movements directed at the injection site. The total number of scratching bouts was quantified from video recordings by a blinded researcher. All procedures adhered to institutional guidelines for animal care and use.

### 4.7. DNFB-Induced Atopic Dermatitis Model

Eight-week-old male ICR mice obtained from Koatech (Gyeonggi-do, Republic of Korea) were acclimated for one week prior to sensitization. On day 7, the dorsal skin was shaved, and 0.15% DNFB was applied four times on days 10, 13, 20, and 27. To evaluate its therapeutic efficacy, PCG (3 mg/kg) was administered daily via intraperitoneal injection from day 10 to day 27. Scratching bouts were counted immediately after DNFB application.

### 4.8. Histological Analysis of Skin Tissue

Dorsal skin tissues were collected from the mice on the final day of the experiment, fixed in 10% neutral-buffered formalin, and embedded in paraffin. Skin sections (4 µm thick) were sliced and mounted on slides. After deparaffinization, the sections were stained with H&E or toluidine blue and examined under a Nikon Eclipse 80i microscope (Tokyo, Japan) at 100× magnification.

### 4.9. Measurement of Serum TSLP Levels by ELISA

The levels of TSLP were measured using a mouse TSLP ELISA kit (catalog #MTLP00; R&D Systems, Minneapolis, MN, USA) according to the manufacturer’s protocol.

### 4.10. Primary Culture and Calcium Imaging of DRG Neurons

Mouse DRG neurons were collected and incubated in Neurobasal^®^ Medium (Gibco, Thermo Fisher Scientific, Waltham, MA, USA) with 1 mg/mL collagenase type II (Worthington Biochemical, Lakewood, NJ, USA) for 40 min at 37 °C in a shaking incubator (60 rpm), followed by 40 min incubation with 2.5 mg/mL trypsin (Gibco, Thermo Fisher Scientific, Waltham, MA, USA) in HBSS (Gibco, Thermo Fisher Scientific, Waltham, MA, USA). After centrifugation (30× *g*, 10 min), cells were resuspended in Neurobasal^®^ Medium supplemented with 10% FBS, 50–100 ng/mL NGF (Invitrogen, Gaithersburg, MD, USA), and 100 U/mL ZellShield^®^ (Minerva BioLabs, Berlin, Germany), then plated in poly-L-lysine-coated 8-well chambers (Lab-Tek, Naperville, IL, USA). Cells were incubated for 2 days at 37 °C in 5% CO_2_ and 95% relative humidity. Calcium imaging was performed using Fluo-3/AM (5 µM; Invitrogen, Carlsbad, CA, USA) mixed with 0.1% Pluronic F-127 in normal buffer solution (NBS: 140 mM NaCl, 5 mM KCl, 2 mM CaCl_2_/EDTA, 0.5 mM MgCl_2_, 10 mM glucose, and 5.5 mM HEPES, pH 7.4), and loaded into the cells for 60 min at 37 °C. After washing with NBS, fluorescence changes were recorded using ECLIPSE Ti-U (Nikon, Tokyo, Japan) or DMi8 inverted microscope (Leica Microsystems Ltd., Wetzlar, Germany). Excitation and emission wavelengths were 488 nm and 515 nm, respectively. Intracellular calcium levels were expressed as the F/F_0_ ratio, where F represents the fluorescence intensity at a given time point, and F_0_ denotes the baseline fluorescence intensity at time 0 s. Analysis was performed using ImageJ software version 1.53t (NIH, Bethesda, MD, USA).

### 4.11. Data and Statistical Analysis

All experiments were conducted independently in triplicate and performed in a randomized fashion. Data are expressed as mean ± standard error of the mean (SEM). Statistical comparisons were made using unpaired or paired Student’s *t*-tests as appropriate, or one-way analysis of variance (ANOVA) with Tukey’s post hoc test for multiple comparisons. Statistical significance was defined as *p* < 0.05. Dose–response curves were analyzed and fitted using GraphPad Prism version 5.0 (GraphPad Software, San Diego, CA, USA).

## Data Availability

The data presented in this study are available on request from the corresponding author.

## Abbreviations

TSLP	Thymic stromal lymphopoietin
PAR2	Protease-activated receptor 2
GPCR	G protein-coupled receptor
KLK	Kallikrein
PCG	Punicalagin
PAR2-AP	PAR2-activating peptide
DNFB	2,4-dinitrofluorobenzene
HaCaT	Human keratinocyte
TRY	Trypsin
TRB	Thrombin
IL-8	Interleukin-8
PAR2-KO	PAR2 knockout
HDF	Human dermal fibroblast
DRG	Dorsal root ganglion
H&E	Hematoxylin and eosin
